# Change and improvement 50 years in the making: a scoping review of the use of soft systems methodology in healthcare

**DOI:** 10.1186/s12913-020-05929-5

**Published:** 2020-11-23

**Authors:** Hanna Augustsson, Kate Churruca, Jeffrey Braithwaite

**Affiliations:** 1 Centre for Healthcare Resilience and Implementation Science, Australian Institute of Health Innovation, Macquarie University, Level 6, 75 Talavera Rd, North Ryde, Sydney, NSW 2109 Australia; 2 Procome research group, Medical Management Centre, Department of Learning, Informatics, Management and Ethics, Karolinska Institutet, Stockholm, Sweden

**Keywords:** Soft systems methodology, Healthcare, Change management, Participation, Collaboration, Stakeholders

## Abstract

**Introduction:**

Improving the quality of healthcare has proven to be a challenging task despite longstanding efforts. Approaches to improvements that consider the strong influence of local context as well as stakeholders’ differing views on the situation are warranted. Soft systems methodology (SSM) includes contextual and multi-perspectival features. However, the way SSM has been applied and the outcomes of using SSM to stimulate productive change in healthcare have not been sufficiently investigated.

**Aim:**

This scoping review aimed to examine and map the use and outcomes of SSM in healthcare settings.

**Method:**

The review was based on Arksey and O’Malley’s framework. We searched six academic databases to January 2019 for peer-reviewed journal articles in English. We also reviewed reference lists of included citations. Articles were included if they were empirical studies focused on the application of SSM in a healthcare setting. Two reviewers conducted the abstract review and one reviewer conducted the full-text review and extracted data on study characteristics, ways of applying SSM and the outcomes of SSM initiatives. Study quality was assessed using Hawker’s Quality Assessment Tool.

**Result:**

A total of 49 studies were included in the final review. SSM had been used in a range of healthcare settings and for a variety of problem situations. The results revealed an inconsistent use of SSM including departing from Checkland’s original vision, applying different tools and involving stakeholders idiosyncratically. The quality of included studies varied and reporting of how SSM had been applied was sometimes inadequate. SSM had most often been used to understand a problem situation and to suggest potential improvements to the situation but to a lesser extent to implement and evaluate these improvements.

**Conclusion:**

SSM is flexible and applicable to a range of problem situations in healthcare settings. However, better reporting of how SSM has been applied as well as evaluation of different types of outcomes, including implementation and intervention outcomes, is needed in order to appreciate more fully the utility and contribution of SSM in healthcare.

**Supplementary Information:**

The online version contains supplementary material available at 10.1186/s12913-020-05929-5.

## Strengths and limitations of this study

- The review was conducted in accordance with Arksey and O’Malley’s framework for scoping reviews and the Preferred Reporting Items for Systematic reviews and Meta-Analyses extension for Scoping Reviews (PRISMA-ScR) guidelines.
- The scoping methodology allowed information from a broad range of studies, using different designs and methods, to be included and synthesised.
- The findings highlight gaps and future directions for research on the use of soft systems methodology in healthcare.
- The review was limited to peer-reviewed articles and English-speaking literature.
- Although it provides insights into use, the review does not provide a definitive account of the effectiveness of soft systems methodology.

## Background

Despite longstanding efforts to improve the quality of healthcare, increasingly there is a recognition of diminishing returns despite the considerable investments made [1]. This is because many of the most pressing issues in healthcare improvement—including integration of services [2, 3], culture change [4], and treating patients with chronic diseases [5]—resist easy solutions. These are all examples of messy, multi-faceted “wicked” problems in that they “have incomplete, contradictory, and changing requirements and complex interdependencies that are often unique to the local setting of the problem” [6]. Multiple stakeholders are involved in wicked problems and typically have very different understandings of the situation as well as its solution.

It is not just the problems that are complex; healthcare systems are also increasingly understood as complex adaptive systems in that they are comprised of diverse, interconnected agents (e.g., patients, clinicians, hospital managers, government policymakers) whose localised interactions over time give rise to emergent, system-level behaviours [7, 8]. Such systems are considered to be more than the sum of their parts; although we may be able to discern patterns in the behaviour and performance of the healthcare system as a whole, the agents that comprise it always inevitably act locally, and are interdependent with those around them. Trying to change or improve some aspect of the system, then, must begin with consideration of this local context [9].

In complexity thinking, many of the traditional, step-wise, scientific approaches to problem solving and engendering change, which focus on breaking problems into discrete parts, implementing standardised solutions, and controlling for “confounding” (i.e., contextual) variables [10, 11], are not effective. Now more than ever, approaches to healthcare improvement are needed that recognise the complexity of context, as well as the varying interests and understandings of a problem held by different stakeholders.

Soft systems methodology (SSM) [12, 13] is an approach to tackling wicked problems and developing action to improve a challenging situation. It relies on system thinking and recognises different perspectives of actors involved in change. The development of SSM began 50 years ago in the 1970s, and an early version of the methodology included the following seven stages: (1) Problem situation considered problematic; (2) Problem situation expressed; (3) Root definitions of relevant purposeful activity system; (4) Conceptual models of the systems named in the root definitions; (5) Comparison of models and real world; (6) Changes: systematically desirable and culturally feasible; (7) Action to improve the problem situation [14]. The seven stage model developed into a two stream model which emphasised that SSM needed to account for both a logic based analysis, i.e,. analysing the tasks included in the situation and a cultural analysis, i.e., assessing the social and political culture influencing the situation [13]. The methodology was later further refined into a less prescriptive four-activity process. The first activity, (1) *Finding out about the problematic situation, including cultural and political dimensions,* aims to gain an overview and insight of the situation in which the problem exists. To facilitate this the methodology involves the development of a rich picture of the situation, which outlines the interconnections between actors, structures and processes involved. The second activity, (2) *Formulating relevant purposeful activity models,* involves the development of conceptual models structuring how activities in the situation could look. This is not intended to be a perfect model to implement but rather function as an aid in structuring the discussion about feasible and desirable changes that could be made to the problematic situation. The third activity, (3) *Debating the situation using the models,* aims to find feasible and desirable changes to implement. In the fourth activity, (4) *Taking action to bring about improvement,* the results from the three previous activities, i.e., the changes decided on, are tested and implemented as a basis for further learning.

The methodology has several tools to aid the improvement process and involves its own language and terms, which are outlined in Table 1.

**Table 1:** Glossary of tools, terms and acronyms used in SSM

**Rich picture –** An illustrative picture of the structures, processes and actors involved in the problem situation and the interdependencies between these.	
**Purposeful activity model (PAM)** – A conceptual model for one or more aspects of the problematical situation outlining a set of purposeful activities relevant to the situation. The model is a set of linked activities that together makes up a purposeful whole.	
**Root definition** – A statement describing the human activity system to be modelled.	
**CATWOE** –A mnemonic reminder to consider the following information about the human activity system:	
**C**ustomers –The beneficiaries or victims affected by the problematical situation and the improvement intervention.	
**A**ctors –The individuals involved in the situation and in performing the improvement intervention.	
**T**ransformation – The change process.	
**W**orldview – Underlying assumptions that makes the improvement intervention worthwhile and important.	
**O**wners – The actors that are responsible for the improvement intervention and who decide whether it will be implemented or not.	
**E**nvironmental constraints and enablers – The contextual factors that may influence the problematical situation and the improvement intervention.	
**The PQR-formula** – A formula useful for defining the root definition. It is applied by answering the questions: what should be done (P), how it should be done (Q) and why it should be done (R).	
**Five E’s** – Criteria for assessing the outcomes of the improvement intervention, including:	
**E**fficacy – does the intervention produce the intended outcomes?	
**E**fficiency – is the improvement being achieved with minimum use of resources?	
**E**ffectiveness – does the intervention help achieve some higher-level or longer-term aim?	
**E**thicality – is the intervention morally appropriate?	
**E**legance – is it an aesthetically pleasing intervention?	

Explanations are based on Checkland and Poulter [12] but interpreted by us and adapted to a language more often used in relation to implementation and improvement science.

Although SSM has been around since the 1970s with modifications through the 1980s and 1990s [15], and there is evidence of its adoption in healthcare research [16], the extent and nature of its use in care settings has not been sufficiently studied. Two previous reviews showed that SSM had been applied in many different areas, including healthcare [17, 18]. One of these reviews focused on the methodological aspects of using SSM in healthcare and found that SSM had been used in different ways including in combination with other methods and modified [17]. The review included both empirical and theoretical papers and provided a brief overview of how SSM has been used in healthcare but limited its search to one data base and did not go into detail about how SSM has been applied [17]. The other review focused on providing a categorisation of fields of application of SSM publications, i.e., categorising papers into empirical and theoretical papers and thereafter a subdivision into type of organisations and type of organisation processes in which SSM has been used [18]. However, these previous reviews have not investigated how SSM has been applied, including the extent of stakeholder involvement in the SSM process, the reasons for using SSM and what outcomes there are in using SSM in healthcare. The aim of this scoping review was to examine and map the use and outcomes of SSM in the context of healthcare and provide an up to date account of the use of SSM in healthcare, focusing on empirical studies. Specific research questions addressed by this review were:

1. In which countries and healthcare settings has SSM been embraced?
2. How has SSM been applied, for example, for problem structuring, or for proposing or implementing interventions?
3. For what type of problem situations, i.e., the area in need of improvement, has SSM been used?
4. To what extent have stakeholders, i.e., the individuals that are affected by or involved in the problem situation and/or improvements that are the focus of the study, been involved and consulted in the SSM process? This includes the number of stakeholder groups that have been involved and the nature of stakeholder involvement, e.g., have stakeholders been actively involved in the SSM process, for instance in creating rich pictures of the problem situation and developing root definitions and PAMs, have stakeholders informed the SSM process by acting as informants in a researcher led study or have stakeholders not been involved at all?
5. What kinds of interventions have been implemented using SSM?
6. What kinds of outcomes have been reported following the use of SSM?

## Method

A scoping review methodology was chosen because it lends itself to mapping relevant literature in a field of interest rather than focusing on a narrow research question [19]. It also allows for studies using different designs and methods to be included and synthesised, which was considered necessary for this review. We followed the methodological stages outlined by Arksey and O’Malley [19] to conduct the review. These are: 1. Identifying the research question, 2. Identifying relevant studies, 3. Study selection, 4. Charting the data, and 5. Collating, summarizing and reporting the results. A protocol for the scoping review has been published [20]. The reporting of the review follows the PRISMA-ScR Checklist [21] (Additional file 1.).

### Eligibility criteria

Citations were assessed against the following inclusion criteria: English-language, peer-reviewed, empirical research articles published in scholarly journals where the full text was available. Using the PCC mnemonic (Population/problem, Concept, Context), recommended for scoping reviews [22], we specified that the content of the citations had to be focused on the application of SSM (concept) in a healthcare setting (context), including policy, primary care, mental health, hospital care, residential aged care, rehabilitation and community health facilities. We did not specify a population since we were interested in the broad application of SSM in healthcare. Studies claiming to apply one or several elements from SSM were included even if SSM had not been applied in its entirety, as were studies using SSM, or aspects of SSM, in combination with other methods. Citations focusing on the use of SSM in settings other than healthcare, e.g., educational settings (including healthcare education) were excluded. Citations without a description of the type of data that was collected and how this data was accessed/collected were excluded. This helped enable us to answer questions about the application of SSM in healthcare. No date limit was applied.

### Information sources

The focus of the review was on peer-reviewed literature and electronic databases from different disciplines such as biomedicine, psychology, health services research, and nursing were searched to identify relevant studies. The databases searched were: Scopus, MEDLINE, Web of Science, CINAHL, EMBASE and PsycINFO.

The search strategy (Table 2) used the term “soft systems method*” to identify citations referring to SSM. Search terms were used to limit the search to the healthcare context, e.g., health* and “acute care”. The wildcard character represents one or more other characters which allows variable endings of keywords, e.g., healthcare, health system, healthcare organisation. In addition to the database search the reference lists from the included citations were snowball-searched to identify additional citations. To reduce the likelihood that relevant articles were overlooked we also hand searched reference lists of key review papers [17, 18]. Search terms were discussed by the three authors and the database searches were then conducted by one researcher (HA) and included all citations published before January 24, 2019. An example of a search strategy is presented in Table 2.

**Table 2:** Search strategy used in Web of Science

TOPIC: ((“soft systems method*”) and (health* or hospital or “acute care” or “primary care” or “general practice” or “aged care” or “nurs* home” or medic* or clinic* or nurs*))	
Refined by: LANGUAGES: (ENGLISH)	
Timespan: All years. Indexes: SCI-EXPANDED, SSCI, A&HCI, CPCI-S, CPCI-SSH, ESCI.	

### Selection of sources of evidence

After duplicates had been removed, all references were imported into Rayyan, a web-based and mobile app that organises and facilitates the initial screening of titles and abstracts [23]. Two reviewers (HA, KC) applied the inclusion and exclusion criteria to a random sample of 20 citations to test the criteria, i.e., piloting the criteria, and to ensure consensus on included citations. Interrater-agreement between the two reviewers was calculated using Cohen’s kappa [24] and yielded an excellent agreement rate (0.88). The two reviewers then, in parallel, applied the inclusion and exclusion criteria on all remaining citations. The citations where the reviewers did not agree on inclusion (*n* = 31) were discussed until consensus was reached. In the next step, one of the reviewers (HA) assessed the full-texts of the included citations for final inclusion. All citations that were given an exclusion decision were also assessed by the second reviewer in order to confirm the decision to exclude the citation.

### Data charting process and data items

An electronic data charting form was developed in excel to guide data charting from included citations. Data concerning study characteristics, e.g., authors, year of publication, and the methodology, e.g., design and data collection as well as information related to the objectives of the review, i.e., how SSM had been applied in healthcare and the outcomes of using SSM were charted (Table 3). The data charting form was piloted by the two reviewers on a selection of the citations to test if the data charting form covered all study objectives and was specific enough to ensure that the reviewers extracted the same data. The pilot test resulted in minor changes to the form. One of the reviewers (HA) then independently charted the data from the remaining citations. Uncertainties were discussed by the two reviewers.

**Table 3:** Overview of data items

Charted data items	
a.	Author(s)
b.	Publication year
c.	Title
d.	Aim
e.	Country of origin
f.	Type of healthcare setting(s)
g.	Methods (design, data collection methods, number and type of participants)
h.	Way of using SSM (for problem structuring, for proposing interventions, for implementing and/or evaluating interventions)
i	Type of problem that SSM has been applied to
j.	Degree of stakeholder participation (i.e., number of stakeholder groups that have been consulted in the different SSM activities and how stakeholders were involved)
k.	Type of intervention implemented (if applicable)
l.	Type of outcomes reported (if applicable)

### Critical appraisal of individual sources of evidence

A critical appraisal of the studies was conducted using the Hawker tool [25]. This tool was developed to systematically review research from different paradigms, i.e., both qualitative and quantitative studies and provide an assessment ranging from very poor to good for a range of domains: abstract and title; introduction and aims; method and data; sampling; data analysis; ethics and bias; results; transferability; and implications and practice. Based on the ratings an overall quality rating of low, medium or high was assigned to each study based on Lorenc et al. [26]. The appraisal was not used to exclude studies from the review but instead added to the information about the quality of reporting of studies using SSM in healthcare. The quality appraisal was conducted by one reviewer (HA) and when questions occurred, these were discussed between the two reviewers.

### Synthesis of results

Results were synthesised and presented using frequency counting as well as summarised in text. The data were compared and synthesised to summarise study characteristics, use of SSM, type of problems that SSM was applied to, stakeholder participation, types of improvement interventions implemented, and outcomes of these improvements if applicable.

### Patient and public involvement

The study involved no patient or public involvement.

## Results

**Fig. 1:**
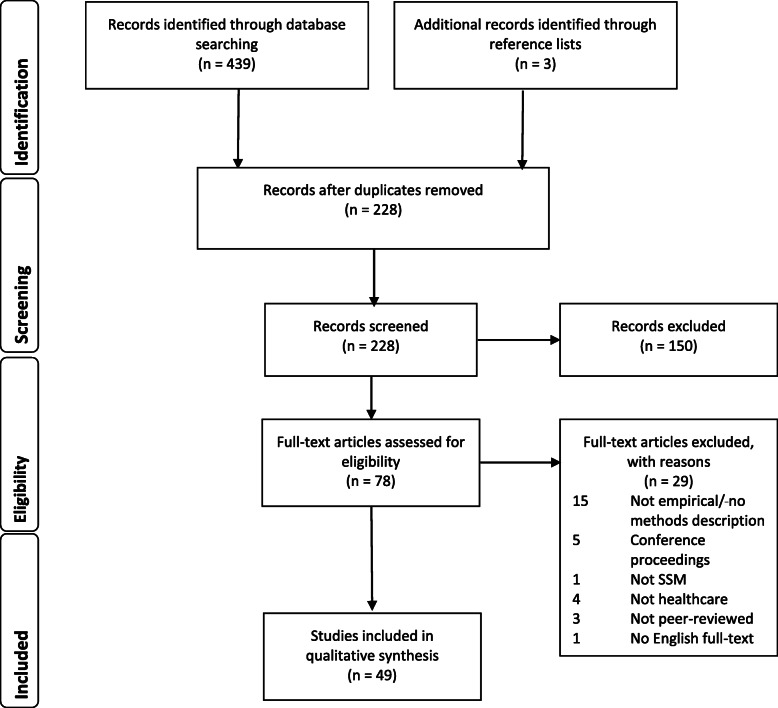
Search and review strategy

The search resulted in 228 unique citations of which 49 were included in the review. Figure 1 demonstrates the inclusion and exclusion of citations at each stage of the screening process, using the PRISMA flow diagram [27].

### Characteristics of studies using SSM in healthcare and quality assessment

The included studies were published between 1986 and 2019, without any clear time trend. A majority of studies originated from the United Kingdom (*n* = 28, 57.1%). Five studies (10.2%) were conducted in the USA, four (8.2%) in Australia and two (4.1%) in Norway. The remainder were single studies conducted in Belgium, Canada, the European union member states, Finland, Greece, Ireland, Portugal, Scotland, South Africa and Turkey.

The included studies were conducted in a wide range of healthcare settings with hospitals (*n* = 15, 30.6%) the most common. Nine studies (18.4%) were conducted in policy settings, five studies (10.2%) in mental health settings and four studies (8.2%) each were held in community settings and multiple types of setting. Other represented settings were aged care (*n* = 2, 4.1%), ambulatory care (*n* = 1, 2%), primary care (*n* = 2, 4.1%), public health (*n* = 2, 4.1%), health informatics management organisations (*n* = 2, 4.1%), ambulance service (*n* = 1, 2%), end-of-life care (*n* = 1, 2%) and blood collection establishments (*n* = 1, 2%).

Two thirds of the studies had a qualitative design (*n* = 34, 69.4%), 14 studies (28.6%) a mixed methods design and one study (2%) was purely quantitative. The majority of studies employed a case study design (*n* = 43, 87.8%). The remaining studies had cross sectional design (*n* = 2, 4.1%), longitudinal design (*n* = 2, 4.1%), or experimental design (*n* = 2, 4.1%).

Interviews were the most commonly used method for collecting data (*n* = 38, 77.6%) followed by observations (*n* = 17, 34.7%), focus group interviews (*n* = 14, 28.6%), and workshops/group discussions (*n* = 14, 28.6%) not characterised as focus groups. The difference between focus groups and other group discussions was not always clear due to a lack of information about their conduct. Other sources of data were surveys (*n* = 8, 16.3%), literature reviews (*n* = 7, 14.3%), document analysis (*n* = 5, 10.2%) and administrative data (*n* = 4, 8.2%).

The studies displayed a wide variety of numbers of data collection methods, ranging between 1 and 6 methods/data sources. Most studies applied one (*n* = 17, 34.7%), two (*n* = 12, 24.5%) or three (*n* = 17, 34.7%) methods. The remaining studies used five (*n* = 2, 4.1%) and six (*n* = 1, 2%) methods.

The quality assessment revealed that 22 studies (44.9%) were of high quality, 21 studies (42.9%) were medium quality and six studies (12.2%) had a low quality when it came to reporting. Table 4 provides an overview of the characteristics of the studies and descriptions of each included study can be found in Additional file 2.

**Table 4:** Study characteristics

	Number of studies	%
**Healthcare setting**		
Hospital	15	30.6
Policy	9	18.4
Mental health	5	10.2
Community	4	8.2
Multiple settings	4	8.2
Aged care	2	4.1
Primary care	2	4.1
Public health	2	4.1
Health informatics management organisations	2	4.1
Ambulatory care	1	2
Ambulance service	1	2
End-of-life care	1	2
Blood collection establishments	1	2
**Method**		
Qualitative	34	69.4
Mixed methods	14	28.6
Quantitative	1	2
**Study design**		
Case study	43	87.7
Cross-sectional	2	4.1
Longitudinal	2	4.1
Experimental design	2	4.1
**Data collection method**^**a**^		
Interviews	38	77.6
Observations	17	34.7
Focus groups interviews	14	28.6
Workshops/group discussions	14	28.6
Survey	8	16.3
Literature review	7	14.3
Document analysis	5	10.2
Administrative data	4	8.2
**Number of data collection methods used**		
1	17	34.7
2	12	24.5
3	17	34.7
4	–	–
5	2	4.1
6	1	2
**Quality assessment**		
High quality	22	44.9
Medium quality	21	42.9
Low quality	6	12.2

^**a**^Percentage exceeds 100 because some studies used multiple data collection methods

### Stakeholder involvement

SSM is a participative approach emphasising the importance of considering all relevant stakeholders’ views and opinions about a situation. The extent of stakeholder involvement differed in the included studies. The number of consulted stakeholder groups varied from none in a study based on existing population data [28] to 20 [29]. A categorisation of the different stakeholders involved showed that the majority of studies (*n* = 35, 71.4%) involved healthcare professionals such as nurses, physicians, pharmacists and dieticians. Healthcare managers, e.g., department managers, were also often involved in the SSM process (*n* = 21, 42.9%). Fourteen studies each (28.6%) included service users or representatives, i.e., patients, family members and patient representatives, and policy makers or administrators. Eleven studies involved staff with an administrative role or support function, e.g., knowledge and information management. Six studies (12.2%) included research and development staff (Table 5).

**Table 5:** Stakeholder involvement

	Number of studies	%
**Number of stakeholder groups involved**		
0–3	13	26.5
4–7	13	26.5
8–11	10	20.4
12–20	4	8.2
Not clear/not stated	9	18.4
**Categories of stakeholders involved**^**a**^		
Healthcare professionals	35	71.4
Healthcare managers	21	42.9
Service users/representatives	14	28.6
Policy makers/administrators	14	28.6
Administrative/support staff	11	22.4
Research and development staff	6	12.2
Not clear/not reported	6	12.2
**Stakeholder involvement category**		
Category 1: Stakeholders involved in the SSM process	21	42.9
Category 2: Stakeholders involved as informants and SSM process conducted by researchers	22	44.9
Category 3: Not clear/not stated	5	10.2
Category 4: No stakeholder involvement	1	2

^**a**^Percentage exceeds 100 because many studies involved multiple stakeholder categories

The studies also illustrate different ways of involving stakeholders in the SSM process. We allocated types of stakeholder involvement into four categories (Table 5): *Category 1*. 21 studies (42.9%) involved stakeholders directly in the SSM process. This category included studies where stakeholders were active in at least parts of the SSM process, such as creating rich pictures or developing PAMs. *Category 2*. 22 studies (44.9%) involved stakeholders as informants/sources of data. This category, which included studies where stakeholders participated as informants, e.g., in interviews, and researchers used the information to inform the researcher led SSM process. *Category 3*. Included five studies (10.2%) where stakeholder involvement was not clear or not stated. This pertained to studies where the reporting of the data collection process was not described in such a way that the type of participation could be decided. *Category 4*. Included one study (2%) based on pre-existing data, which therefore did not include any stakeholders.

### Ways of using SSM

The purpose of using SSM varied with the majority (*n* = 20, 40.8%) harnessing SSM both for problem structuring and for proposing interventions or recommendations for improvements. Eight studies (16.3%) used SSM solely for problem structuring without proposing any interventions or recommendations. Five studies (10.2%) used SSM for implementing interventions or making recommendations in addition to problem structuring and proposing interventions or recommendations. The remaining studies utilised SSM for evaluating interventions (*n* = 6, 12.2%), for determining objectives for simulation studies (*n* = 2, 4.1%), to describe or understand models of care or processes in healthcare (*n* = 4, 8.2%) or for other purposes (*n* = 4, 8.2%) (Table 6).

**Table 6:** Ways of using SSM

	Number of studies	%
**Purpose of using SSM**		
Problem structuring and proposing improvements	20	40.8
Problem structuring	8	16.3
Evaluation	6	12.2
Problem structuring, proposing and implementing improvements	5	10.2
Describing or understanding models of care or processes in healthcare	4	8.2
Determine objectives for a simulation study	2	4.1
Other	4	8.2
**Problem situation/reason for using SSM**		
Health system improvement	9	18.4
Care process improvement	8	16.3
Policy improvement	7	14.3
Information system development/improvement	6	12.3
Describe/improve knowledge management system	3	6.1
Intervention/program/care model evaluation	3	6.1
Analyse/improve practice development	3	6.1
Analyse/improve teamwork	2	4.1
Other	8	16.3
**Type of SSM used**		
Seven stage	13	26.5
Four activity	6	12.3
Combined with other method	11	22.5
Part of the method	6	12.3
New adapted version	3	6.1
Two strands	1	2
Nine stage	1	2
Not clear/not stated	8	16.3
**SSM tools applied**^a^		
PAM	33	67.3
Root definition	29	59.2
CATWOE	28	57.1
Rich picture	20	40.1
Comparison of real world and PAM	17	34.7
Three Es	4^b^	8.2
PQR-formula	2	4.1
Five Es	1	2

^a^Percentage exceeds 100 because some studies applied multiple SSM tools

^b^One study used an adapted version of the Three Es [30]

The problem situations to which SSM was applied varied greatly, facilitating a broad categorisation of the different problem situations (Table 6). The most common reason for using SSM was for *health systems improvements* (*n* = 9, 18.4%), for instance to inform the development of integrated health and social services in mental health [31] or to improve inadequate and fragmented services for children with serious emotional disturbance [32]. The second most common reason was to *improve care processes* (*n* = 8, 16.2%) such as enhancing continuity of care for palliative patients in a community setting [33] and reducing long waiting times for patients after having arrived for their appointments [34]. Seven studies (14.3%) used SSM for different kinds of *policy improvements* including contracting in the National Health Service (NHS) in England [35, 36] and making suggestions for the development of a policy for the organization of child and adolescent mental healthcare services in Belgium [37]. Six studies (12.3%) engaged with SSM for *information system development/improvement*. In these studies, SSM was used to develop information systems for particular healthcare settings or for improving an existing information system. One example was the use of SSM to develop an information system that facilitates the sharing of information across stakeholders involved with rehabilitative care for older patients [38]. The remaining studies had applied SSM to *knowledge management improvements* (*n* = 3, 6.1%), for *intervention/program/care model evaluation* (*n* = 3, 6.1%), to *analyse/improve practice development* (*n* = 3, 6.1%), and to *analyse/improve teamwork* (*n* = 2, 4.1%). Eight studies (16.2%) took to SSM for *other purposes* that could not be sorted into any of the categories, e.g., to explore the development of specialist staffing [39] and to examine community partnership, engagement and participation [40]. All the problem situations to which SSM was applied are described in Additional file 2.

The included studies used different versions of SSM. Thirteen studies (26.6%) used the seven-stage version and the four activity version was use in six studies (12.3%). A number of studies (11, 22.5%) used SSM in combination with other methods or used parts of the methodology, e.g., only the CATWOE, (*n* = 6, 12.3%). Three studies (6.1%) used a new adapted version of SSM and one study each (2%) used the two strands model and a nine-stage version of SSM. In eight (16.3%) of the included studies it was not clear what type of SSM was used or in what way SSM had been used.

There was also wide variation in how many and which SSM tools were applied in the studies. The most frequently used tools were PAM (*n* = 33, 67.3%), Root definition (*n* = 29, 59.2%), CATWOE (*n* = 28, 57.1%) and Rich picture (*n* = 20, 40.1%). Seventeen (34.7%) studies described having compared the real world and the PAMs. The three Es (*n* = 4, 8.2%), the five Es (*n* = 1, 2%) and the PQR formula (*n* = 2, 4.1%) were used to a lesser extent (Table 6).

### Outcomes of implemented improvements

Although 20 studies proposed improvements only five studies reported that the improvements had been implemented. These studies reported some, often narrative, information about the outcomes of these improvements. Kotiadis et al. [41] developed a model for how a multidisciplinary team should function. The model was at least partially implemented 3 years later resulting in a better functioning team. However, the authors stressed that it was not possible to determine whether this was because of the SSM intervention, although they adduced some evidence to support that. In the study by Hales and Chakravorty [42] SSM was used together with mindfulness to implement high reliability organization (HRO) principles. SSM facilitated the implementation of HRO, which ultimately led to improved reliability in a critical care unit, including an increase in the percentage of patients discharged alive with stable vital signs. Before HRO implementation, this measure was 93.8% and after implementation, it increased to 99.5%, a statistically significant increase. In the study by Lehaney et al. [34], a procedure to reduce unexpected non-attendance of patients by using simple rules for patient scheduling was implemented. This strategy resulted in reduced clinic waiting times. Pentland et al. [43] used SSM to design and implement improvements to a mental health service, enhancing knowledge acquisition and management activities in order to facilitate effective evidence-based practices. A ten-month change programme resulted in a number of changes to the knowledge acquisition and management structures and procedures. Holm et al. [29], applied SSM to improvements in a central surgery unit (CSU). Several enhancements that would directly and indirectly affect surgical activities were suggested, some of which were implemented. One example was the implementation of more flexibility to the duration of shifts, which was accepted and adopted by staff, resulting in fewer procedures being cancelled due to expected overtime. The authors also discussed that the management and the actors involved in the CSU seemed more aware of the importance of team behaviour and team leadership as well as communication and cooperation between staff groups after the study.

## Discussion

This scoping review provides an overview and synthesis of how SSM has been applied in healthcare. It illustrates a wide area of application of SSM, and highlights the way people have embraced it – with flexibility and adaptability. As a change strategy it has been used across many kinds of problem situations which might be seen as a benefit. But critics might say that SSM has been inconsistently applied – and not routinely in the way that Checkland and colleagues originally envisaged its use. In short, researchers and change agents in healthcare have adapted SSM to suit different contexts, implementation aims and study designs. This means we have witnessed a proliferation of different versions of SSM, application of different tools in divergent studies and involving stakeholders to dissimilar extents. Overarchingly, most studies used SSM for problem structuring and for proposing improvement interventions. However, those who did report SSM use were less likely to report on the implementation and outcomes of their interventions.

The wide area of application shows that SSM has been considered applicable to many different settings and complex problems found in healthcare (e.g., inadequate and fragmented services for children with serious emotional disturbance [32]; the need to increase safe blood management in the EU [44]). SSM has been proposed to be especially suited to these types of wicked problems which may explain the flexible use of SSM in different settings and situations in healthcare. Given how hard it is to make change in an arena that has so many fundamentally complex challenges, the SSM methods and approaches to deal with these complexities are warranted [10, 45]. At the same time, research methods used in healthcare contexts have traditionally been of a positivist nature (e.g., randomised controlled trials, formal before and after or time series studies), yet many researchers and practitioners now acknowledge the need for less linear and more context-sensitive methods [46, 47].

### Stakeholder involvement and co-creation

The review revealed wide variation when it came to involving stakeholders, ranging from no involvement to full involvement in exploring the problem situation, developing PAMs and taking action to improve the situation. Arguments have been made that research and improvement attempts that build on co-creation and collaboration have the potential to generate more relevant, applicable and contextually-sensitive knowledge and thereby deliver enhanced processes and outcomes [48, 49]. With its emphasis on stakeholder involvement baked into the model, SSM has potential to make far-reaching changes. Based on the potential of co-creation, it can be hypothesised that SSM studies that involve stakeholders to a greater extent are more likely to achieve effective outcomes.

### Inconsistent application of SSM

The inconsistent use of SSM, including different versions and different tools applied, makes it difficult to compare the use of SSM across studies. Although the aim of this review was not to determine the effectiveness of SSM, this finding indicates that it may be premature to comment on the effectiveness of SSM for making widespread improvements in healthcare despite the existence of the method for some time. The inconsistent use of SSM across studies also impedes the ability to assess the core components of the methodology, i.e., if certain tools or components of the methodology are especially important for bringing about successful improvements. The varied use of SSM may be related to the fact that SSM is indeed intended to be a flexible approach (originating in the initial work of Peter Checkland and his colleagues) which is also illustrated by the development of the more prescriptive seven-stage version of SSM to the less prescriptive four-activity process [13]. However, the seven stage version was the most commonly applied and newer studies also chose to use this version [e.g., [50]], possibly suggesting that a more prescriptive or multi-staged version may be easier to apply. Yet, use of earlier versions of SSM may contribute to a continued inconsistent use of SSM. It is possible that the flexibility in itself is a core component of SSM. Nevertheless, the way SSM was applied needs to be clearly described to facilitate understanding of the circumstances under which SSM may be useful, and in order to facilitate cross-study comparison.

### Evaluating outcomes of using SSM in healthcare

As we have seen, SSM had been put to work for varied purposes, mostly to understand or structure a problem situation in combination with coming up with proposals for improvement. To a much lesser extent, these solutions were implemented and evaluated. This confirms previous findings from a more selective overview of the use of SSM in healthcare [16]. Possibly this indicates that the strength of SSM is in the problem structuring and modelling phase, including debating a difficult situation where earlier attempts to improve the situation have failed, and coming up with improvement suggestions that can be tested, rather than the process of actually putting these improvements into action, which may require other approaches [16].

One important issue that arises in relation to evaluating the effectiveness of SSM is the question of what types of outcomes to consider. The traditional approach to the evaluation of effectiveness focuses on intervention outcomes, i.e., the outcomes of the improvement intervention implemented as a result of SSM. However, these outcomes will not only depend on the value of SSM but also on how such an intervention is implemented and the nature and content of the improvement intervention itself. This suggests that different types of outcomes need to be considered when evaluating SSM. We propose that evaluations of using SSM in healthcare should include measures of whether the application of SSM led to an understanding about the problem situation (evaluation of the complex system in which the problem exists), whether SSM facilitated the formulation of suggestions for improvements, whether improvements were successfully implemented (implementation outcomes [51]) and whether the improvements led to positive outcomes (intervention outcomes).

The studies included in this review mainly reported outcomes related to the understanding about the problem situations and formulation of potential improvements. Nevertheless, the lack of studies reporting on the implementation and intervention outcomes of using SSM limits the potential to assess the capacity of SSM to evoke change and deliver positive outcomes in healthcare.

In sum, supporting an earlier review of SSM [17], our review revealed an insufficient description of how SSM had been applied and how data had been collected and analysed in several of the studies. This obviously limits the potential to make comparisons across studies as well as reduces the possibility of using previous research success to inform future studies.

### Implications and future directions

Our results point to the need for better reporting of the use of SSM in healthcare. Studies should clearly report on how SSM was used, including which version of SSM that was used, which tools that were applied, how stakeholders were involved, and how data was collected and analysed. The review also revealed a gap in knowledge regarding the outcomes of using SSM. Future studies should strive to clearly report different types of outcomes from using SSM in addition to describing the context of the study and the application of SSM. This will facilitate assessment of under what conditions SSM may be a useful and an effective approach. Furthermore, the varied involvement of stakeholders in the process and the fact that participation lies at the core of the SSM methodology calls for further investigation of how the level of involvement influences the process and the outcomes of SSM.

### Strengths and limitations

The review followed a comprehensive methodology for scoping reviews as outlined by Arksey and O’Malley [19] and the reporting of the study followed the PRSIMA-ScR [21]. A double blinded review of all abstracts was applied to ensure consistency of study inclusion. Full-texts were screened by one reviewer only; however, all exclusion decisions as well as uncertainties were discussed with a second reviewer. The review is limited to studies in English published in the peer review literature, meaning that studies reported in the grey literature or in languages other than English are not covered. A difficulty encountered was the sometimes inadequate methodological description of the studies, which limited the information that could be extracted. As such, important information about how SSM has been used in healthcare may have been missed. For instance, assessment of participation was made based on the information reported in the included papers and the true level of participation may not have been captured in some cases because of insufficient reporting about how stakeholders were involved. This study does not therefore provide a full account of the effectiveness of using SSM in healthcare and it was not possible to assess the impact of level of involvement of stakeholders on the success of the SSM process.

## Conclusion

SSM is flexible and applicable for a range of settings and for a wide array of problem situations in healthcare. SSM has been inconsistently used and the reporting of how SSM is applied is often insufficient, which limits its comparability across studies. Better reporting of how SSM has been applied as well as evaluation of different types of outcomes, including implementation and intervention outcomes, is needed in order to be able to more fully appreciate the usefulness of SSM in healthcare.

## Supplementary Information

**Additional file 1.** Preferred Reporting Items for Systematic reviews and Meta-Analyses extension for Scoping Reviews (PRISMA-ScR) Checklist.

**Additional file 2.** Table of all studies included in the review.

## Data Availability

Not applicable.

## Abbreviations

PAM: Purposeful activity model
PRISMA-ScR: Preferred Reporting Items for Systematic reviews and Meta-Analyses extension for Scoping Reviews
SSM: Soft systems methodology

## References

[1] Braithwaite J (2018). Changing how we think about healthcare improvement. BMJ..

[2] Laugaland K, Aase K (2017). The demands imposed by a health care reform on clinical work in transitional care of the elderly: a multi-faceted Janus. Resilient Health Care.

[3] Shaw SE, Rosen R (2013). Fragmentation: a wicked problem with an integrated solution?. J Health Serv Res Policy.

[4] Strudwick R, Thomas W, Hujala A, Laulainen S, McMurray R (2018). Blame culture in the National Health Service (NHS), UK. The Management of Wicked Problems in health and social care.

[5] Thomas W, Thomas W, Hujala A, Laulainen S, McMurray R (2018). Unpacking dependency; managing ‘becoming’ supporting the experiences of patients living with chronic disease. The Management of Wicked Problems in health and social care.

[6] Periyakoil VS (2007). Taming wicked problems in modern health care systems. J Palliat Med.

[7] Braithwaite J, Churruca K, Ellis LA (2017). Can we fix the uber-complexities of healthcare?. J R Soc Med.

[8] Plsek PE, Greenhalgh T (2001). The challenge of complexity in health care. BMJ..

[9] May CR, Johnson M, Finch T (2016). Implementation, context and complexity. Implement Sci.

[10] Braithwaite J, Churruca K, Long JC, Ellis LA, Herkes J (2018). When complexity science meets implementation science: a theoretical and empirical analysis of systems change. BMC Med.

[11] Hawe P, Shiell A, Riley T (2004). Complex interventions: how “out of control” can a randomised controlled trial be?. BMJ..

[12] Checkland P, Poulter J (2006). Learning for action: a short definitive account of soft systems methodology and its use, for practitioners, teachers and students.

[13] Checkland P (2000). Soft systems methodology: a 30-year retrospective. Syst Res Behav Sci.

[14] Checkland P (1972). Towards a systems-based methodology for real-worls probem solving. J Syst Eng.

[15] Mingers J (2000). An idea ahead of its time: the history and development of soft systems methodology. Syst Pract Action Res.

[16] Augustsson H, Churruca K, Braithwaite J (2019). Re-energising the way we manage change in healthcare: the case for soft systems methodology and its application to evidence-based practice. BMC Health Serv Res.

[17] Powell JH, Mustafee N (2017). Widening requirements capture with soft methods: an investigation of hybrid M&S studies in health care. J Oper Res Soc.

[18] Van de Water H, Schinkel M, Rozier R (2007). Fields of application of SSM: a categorization of publications. J Oper Res Soc.

[19] Arksey H, O'Malley L (2005). Scoping studies: towards a methodological framework. Int J Soc Res Methodol.

[20] Augustsson H, Churruca K, Braithwaite J (2019). Mapping the use of soft systems methodology for change management in healthcare: a scoping review protocol. BMJ Open.

[21] Tricco AC, Lillie E, Zarin W, O'Brien KK, Colquhoun H, Levac D (2018). PRISMA extension for scoping reviews (PRISMA-ScR): checklist and explanation. Ann Intern Med.

[22] The Joanna Briggs Institute. The Joanna Briggs institute reviewers manual 2015: methodology for JBI scoping reviews. Adelaide: The Joanna Briggs Institute; 2015.

[23] Ouzzani M, Hammady H, Fedorowicz Z, Elmagarmid A (2016). Rayyan—a web and mobile app for systematic reviews. Syst Rev.

[24] McHugh ML (2012). Interrater reliability: the kappa statistic. Biochemia Medica.

[25] Hawker S, Payne S, Kerr C, Hardey M, Powell J (2002). Appraising the evidence: reviewing disparate data systematically. Qual Health Res.

[26] Lorenc T, Petticrew M, Whitehead M, Neary D, Clayton S, Wright K, et al. Crime Fear of Crime and Mental Health: Synthesis of Theory and Systematic Reviews of Interventions and Qualitative Evidence. Public Health Res. 2014;2(2):1–398. 25642507

[27] Moher D, Liberati A, Tetzlaff J, Altman DG (2009). Group atP. Preferred reporting items for systematic reviews and meta-analyses: the PRISMA statement. Ann Intern Med.

[28] Cardoso-Grilo T, Monteiro M, Oliveira MD, Amorim-Lopes M, Barbosa-Póvoa A (2019). From problem structuring to optimization: a multi-methodological framework to assist the planning of medical training. Eur J Oper Res.

[29] Holm LB, Dahl FA, Barra M (2013). Towards a multimethodology in health care–synergies between soft systems methodology and discrete event simulation. Health Syst.

[30] Kotiadis K, Tako AA, Vasilakis C (2014). A participative and facilitative conceptual modelling framework for discrete event simulation studies in healthcare. J Oper Res Soc.

[31] Gibb CE, Morrow M, Clarke CL, Cook G, Gertig P, Ramprogus V (2002). Transdisciplinary working: evaluating the development of health and social care provision in mental health. J Ment Health.

[32] Hodges S, Ferreira K, Israel N (2012). "if we're going to change things, it has to be systemic:" systems change in children's mental health. Am J Community Psychol.

[33] Price M, Lau FY (2013). Provider connectedness and communication patterns: extending continuity of care in the context of the circle of care. BMC Health Serv Res.

[34] Lehaney B, Clarke SA, Paul RJ (1999). A case of an intervention in an outpatients department. J Oper Res Soc.

[35] Hindle T (1995). Developing GP monitoring systems guided by a soft systems approach. Health Serv Manag Res.

[36] Burgoyne JG, Brown DH, Hindle A, Mumford MJ (1997). A multi-disciplinary identification of issues associated with 'Contracting' in market-oriented health service reforms. Br J Manag.

[37] Vandenbroeck P, Dechenne R, Becher K, Eyssen M, Van den Heede K (2014). Recommendations for the organization of mental health services for children and adolescents in Belgium: use of the soft systems methodology. Health Policy.

[38] Connell NA, Goddard AR, Philp I, Bray J (1998). Patient-centred performance monitoring systems and multi-agency care provision: a case study using a stakeholder participative approach. Health Serv Manag Res.

[39] Reed J, Inglis P, Cook G, Clarke C, Cook M (2007). Specialist nurses for older people: implications from UK development sites. J Adv Nurs.

[40] Carr SM, Clarke CL, Molyneux J, Jones D (2006). Facilitating participation: a health action zone experience. Prim Health Care Res Dev.

[41] Kotiadis K, Tako AA, Rouwette E, Vasilakis C, Brennan J, Gandhi P (2013). Using a model of the performance measures in soft systems methodology (SSM) to take action: a case study in health care. J Oper Res Soc.

[42] Hales DN, Chakravorty SS (2016). Creating high reliability organizations using mindfulness. J Bus Res.

[43] Pentland D, Forsyth K, Maciver D, Walsh M, Murray R, Irvine L (2014). Enabling integrated knowledge acquisition and management in health care teams. Knowl Manag Res Pract.

[44] Adamides E, Maniatis A (2001). A systems study for a European community program on inspection and accreditation of blood collection establishments. Syst Pract Action Res.

[45] Kitson A, Brook A, Harvey G, Jordan Z, Marshall R, O’Shea R (2018). Using complexity and network concepts to inform healthcare knowledge translation. Int J Health Policy Manag.

[46] Wells M, Williams B, Treweek S, Coyle J, Taylor J (2012). Intervention description is not enough: evidence from an in-depth multiple case study on the untold role and impact of context in randomised controlled trials of seven complex interventions. Trials.

[47] Blackwood B, O'Halloran P, Porter S (2010). On the problems of mixing RCTs with qualitative research: the case of the MRC framework for the evaluation of complex healthcare interventions. J Res Nurs.

[48] Rycroft-Malone J, Burton CR, Bucknall T, Graham ID, Hutchinson AM, Stacey D (2016). Collaboration and co-production of knowledge in healthcare: opportunities and challenges. Int J Health Policy Manag.

[49] Greenhalgh T, Jackson C, Shaw S, Janamian T (2016). Achieving research impact through co-creation in community-based health services: literature review and case study. The Milbank Quarterly.

[50] Emes M, Smith S, Ward S, Smith A, Ming T (2017). Care and flow: using soft systems methodology to understand tensions in the patient discharge process. Health Syst.

[51] Proctor E, Silmere H, Raghavan R, Hovmand P, Aarons G, Bunger A (2011). Outcomes for implementation research: conceptual distinctions, measurement challenges, and research agenda. Admin Pol Ment Health.

