# Supplementary material for: Change and improvement 50 years in the making: a scoping review of the use of soft systems methodology in healthcare
Source: BMC Health Serv Res. 2020 Nov 23;20:1063. doi: 10.1186/s12913-020-05929-5 (PMC7684911; doi:10.1186/s12913-020-05929-5)
Supplement: Supplementary file 2 — Additional file 2. Table of all studies included in the review. [file 12913_2020_5929_MOESM2_ESM.docx]

**Additional file 2.**

**Table of studies included in the review.**

| **Authoryear ref.** | **Setting** | **Problem/reason for using SSM** | **Purpose of using SSM** | **Data collection method** | **Type of SSM** | **Featured SSM tools** | **Type of stakeholder involvement; categories of stakeholders involved** | **Reported outcomes in cases where proposed improvements were implemented** | **Quality score** |
| --- | --- | --- | --- | --- | --- | --- | --- | --- | --- |
| Connell et al 1998[[1](#_ENREF_1)] | Community allied health | Information system development/improvement | Problem structuring | Workshops | Part of the method | Rich picture | Involved in SSM; Healthcare professionals, Service users/representatives, Policy makers/administrators, Community Health Services Information Manager | N/A | Medium |
| Kalim, et al 2006[[2](#_ENREF_2)] | Policy | Policy improvement | Problem structuring and proposing improvements | Interviews | 7 stage | CATWOE, Rich picture, PAM, Root definition, Five Es, Comparison of real world and PAM | Involved as informants; Healthcare professionals, Policy makers/administrators | N/A | Low |
| Clarke et al 2001[[3](#_ENREF_3)] | Mixed | Analyse/improve practice development | Problem structuring | Interviews, focus groups, survey | Part of the method | PAM, Comparison of real world and PAM | Involved as informants; Healthcare professionals, Policy makers/administrators, R&D staff | N/A | High |
| Darzentas et al 1993[[4](#_ENREF_4)] | Primary Care | Information system development/improvement | Problem structuring and proposing improvements | Interviews | Part of the method | Rich picture, PAM, Root definition | Involved as informants; Healthcare professionals, Healthcare managers, Administrative/support staff | N/A | Medium |
| Emes et al 2017[[5](#_ENREF_5)] | Hospital | Care process improvement | Problem structuring and proposing improvements | Interviews, workshop | 7 stage | CATWOE, Rich picture, PAM, Root definition, Comparison of real world and PAM | Involved in SSM; Healthcare professionals, Healthcare managers, Service users/representatives, Administrative/support staff | N/A | High |
| Kotiadis et al 2007[[6](#_ENREF_6)] | Community allied health | Health system improvement | Determine objectives for a simulation study | Interviews, workshop | 4 activity model | CATWOE, PAM, Root definition, Three Es | Not stated | N/A | Medium |
| Kotiadis et al 2013[[7](#_ENREF_7)] | Hospital | Analyse/improve teamwork | Problem structuring, proposing and implementing improvements | Interviews, observation | New adapted version | CATWOE, PAM, Root definition, Three Es | Involved in SSM; Healthcare professionals, multidisciplinary team coordinator | A model for how a multidisciplinary team should function was developed and at least partially implemented. The team functioned better three years after the first SSM workshop but it was not possible to determine whether this was because of the SSM intervention although some evidence supported that. | High |
| Kotiadis et al 2014[[8](#_ENREF_8)] | Hospital | Health system improvement | Determine objectives for a simulation study | Observations, workshop | Combined with other method | CATWOE, PAM, Root definition, Three Es* | Involved in SSM; Healthcare professionals, Healthcare managers | N/A | Medium |
| Sinclair et al 2014[[9](#_ENREF_9)] | Multiple settings | Health system improvement | Problem structuring and proposing improvements | Interviews, focus groups, observations, document analysis, workshop, administrative data | 7 stage | CATWOE, Rich picture | Involved in SSM; Healthcare professionals, Service users/representatives, Policy makers/administrators | N/A | High |
| Wells et al 1995[[10](#_ENREF_10)] | Mental health | Analyse/improve practice development | Problem structuring and proposing improvements | Interviews, observations, document analysis | 7 stage | CATWOE, Rich picture, PAM, Root definition, Comparison of real world and PAM | Involved as informants; Healthcare professionals, Healthcare managers | N/A | Medium |
| Thomas et al 2014[[11](#_ENREF_11)] | Hospital | Describe and analyse context for an intervention | Evaluation | Focus groups | Combined with other method | CATWOE | Involved as informants; Healthcare professionals, Healthcare managers, Administrative/support staff | N/A | High |
| Torlak et al 2014[[12](#_ENREF_12)] | Hospital | Health system improvement | Problem structuring and proposing improvements | Interviews | The two strands (or streams) model | CATWOE, Rich picture, PAM, Root definition, Three Es. Comparison of real world and PAM | Involved in SSM; Healthcare professionals, Healthcare managers, members of the Private Hospitals and Health Institutions Association | N/A | Medium |
| Luckett et al 2003[[13](#_ENREF_13)] | Policy | Policy improvement | Problem structuring and proposing improvements | Interviews | Combined with other method | CATWOE, PAM, Root definition | Involved in SSM; Healthcare managers, Policy makers/administrators, R&D staff | N/A | Medium |
| Brown 1997[[14](#_ENREF_14)] | Policy | Policy improvement | Problem structuring and proposing improvements | Interviews | 7 stage | CATWOE, PAM, Root definition, Comparison of real world and PAM | Involved in SSM; Policy makers/administrators | N/A | Medium |
| Burgoyne et al 1997[[15](#_ENREF_15)] | Policy | Policy improvement | Describing or understanding models of care or processes in healthcare | Interviews | 7 stage | PAM | Involved as informants; Healthcare professionals, Policy makers/administrators | N/A | Medium |
| Carr et al 2006[[16](#_ENREF_16)] | Policy | Examine community partnership, engagement and participation | Evaluation | Interviews | Combined with other method | Not stated | Involved as informants; Not stated | N/A | High |
| Carr et al 2009[[17](#_ENREF_17)] | Public health | Intervention/program/care model evaluation | Evaluation | Interviews, observations, survey | Combined with other method | Not stated | Involved as informants; Healthcare managers, managers from other sectors | N/A | Medium |
| Carter et al 2019[[18](#_ENREF_18)] | Hospital | Intervention/program/care model evaluation | Evaluation | Interviews, observations, survey | Part of the method | CATWOE, Root definition | Involved as informants; Healthcare professionals, Service users/representatives | N/A | High |
| Clarke et al 2002[[19](#_ENREF_19)] | Multiple settings | Analyse/improve practice development | Describing or understanding models of care or processes in healthcare | Interviews, focus groups | Not clear | Not stated | Involved as informants; Healthcare professionals, Healthcare managers, Policy makers/administrators, R&D staff | N/A | Medium |
| Cook et al 2001[[20](#_ENREF_20)] | Community allied health | Analyse/improve teamwork | Evaluation | Interviews, focus groups, workshop | Not clear | Not stated | Not stated; Healthcare professionals, Healthcare managers | N/A | Medium |
| Crowe et al 2017[[21](#_ENREF_21)] | Multiple settings | Care process improvement | Problem structuring and proposing improvements | Interviews, literature review, administrative data | 4 activity model | CATWOE, Rich picture, PAM, Root definition | Involved in SSM; Healthcare professionals, Service users/representatives, R&D staff | N/A | High |
| Fennessy et al 2001[[22](#_ENREF_22)] | Hospital | Describe/improve knowledge management system | Problem structuring | Interviews, focus groups, observations | Not clear | CATWOE, Rich picture, Root definition | Involved as informants; Administrative/support staff | N/A | Low |
| Gibb et al 2002[[23](#_ENREF_23)] | Mental health | Health system improvement | Evaluation | Interviews, focus groups, workshop | Not clear | Not stated | Involved in SSM; Healthcare professionals, Policy makers/administrators, Administrative/support staff, Carer’s Centre Development Worker | N/A | Medium |
| Gillies et al 2008[[24](#_ENREF_24)] | Health informatics management organisation | Describe/improve knowledge management system | Problem structuring and proposing improvements | Focus groups, survey, administrative data | 7 stage | CATWOE, Rich picture, PAM, Root definition, Comparison of real world and PAM | Involved as informants; Administrative/support staff | N/A | Medium |
| Gillies et al 2009[[25](#_ENREF_25)] | Health informatics management organisation | Information system improvement | Problem structuring and proposing improvements | Interviews, survey, literature review | 7 stage | CATWOE, Rich picture, PAM, Root definition, Comparison of real world and PAM | Involved as informants; Healthcare professionals, Administrative/support staff | N/A | Medium |
| Hales et al 2016[[26](#_ENREF_26)] | Hospital | Health system improvement | Problem structuring, proposing and implementing improvements | Interviews, observations, document analysis | 7 stage | CATWOE, PAM, Root definition | Involved in SSM; Healthcare professionals, Healthcare managers, Service users/representatives, Policy makers/administrators | SSM and mindfulness facilitated implementation of High Reliability Organization and performance at the critical care unit was improved, including an increase in the percentage of patients discharged alive with stable vital signs. | High |
| Hindle 1995[[27](#_ENREF_27)] | Policy | Policy improvement | Problem stucturing | Interviews | Not clear | PAM | Involved in SSM; not stated | N/A | Low |
| Hindle et al 1995[[28](#_ENREF_28)] | Policy | Policy improvement | Problem structuring and proposing improvements | Interviews, workshop | Part of the method | CATWOE, PAM, Root definition | Involved in SSM; not stated | N/A | Medium |
| Hodges et al 2012[[29](#_ENREF_29)] | Mental health | Health system improvement | Developing a conceptual model for systems change for systems of care | Interviews, observations, document analysis | Not clear | PAM | Involved in SSM; Healthcare professionals, Healthcare managers, Service users/representatives, Policy makers/administrators | N/A | Medium |
| Kotiadis et al 2006[[30](#_ENREF_30)] | Aged Care | Intervention/program/care model evaluation | Problem structuring | Interviews, observations, workshop | Combined with other method | CATWOE, PAM, Root definition | Not stated | N/A | Low |
| Lauri 1992[[31](#_ENREF_31)] | Primary Care | Describe the development and testing of a computer simulation program to asses decision making in child health care | Simulation of public health nurses decision making in child healthcare | Completion of simulation scenarios | Not clear | Not stated | Not stated; Healthcare professionals | N/A | High |
| Lehaney et al 1999[[32](#_ENREF_32)] | Hospital | Care process improvement | Problem structuring, proposing and implementing improvements | Workshop | Combined with other method | CATWOE, Rich picture, PAM, Root definition | Involved in SSM; Healthcare professionals, Healthcare managers, Policy makers/administrators | A procedure to reduce unexpected non-attendance of patients was implemented and reduced in-clinic waiting times. | High |
| Mukotekwa et al 2007[[33](#_ENREF_33)] | Hospital | Care process improvement | Problem structuring and proposing improvements | Interviews | 7 stage | CATWOE, Rich picture, PAM, Root definition | Involved as informants; Healthcare professionals, Healthcare managers, Service users/representatives |  | High |
| Newell et al 2017[[34](#_ENREF_34)] | Hospital | Care process improvement | Problem structuring and proposing improvements | Interviews, focus groups | 7 stage | CATWOE, Root definition | Involved in SSM; Healthcare professionals | N/A | Medium |
| O'Meara 2003[[35](#_ENREF_35)] | Ambulance service | Health system improvement | Problem structuring and proposing improvements | Interviews, focus groups, observations, survey, literature review | 4 activity model | PAM, Comparison of real world and PAM | Involved as informants; Healthcare professionals, Healthcare managers, Service users/representatives | N/A | Medium |
| Price et al 2013[[36](#_ENREF_36)] | End of life care | Care process improvement | Problem structuring and proposing improvements | Interviews, workshop | New adapted version | Rich picture, PAM | Involved as informants; Healthcare professionals | N/A | High |
| Reed et al 2007[[37](#_ENREF_37)] | Aged Care | Explore the development of specialist staffing | Explore the development of specialist staffing | Interviews | Not clear | Not stated | Involved as informants; Healthcare professionals, Healthcare managers, Service users/representatives, R&D staff | N/A | High |
| Unertl et al 2009[[38](#_ENREF_38)] | Ambulatory care | Information system development/improvement | Describing or understanding models of care or processes in healthcare | Interviews, observations | Combined with other method | Rich picture | Involved as informants; Administrative/support staff | N/A | High |
| Wells 2006[[39](#_ENREF_39)] | Mental health | Health system improvement | Problem structuring and proposing improvements | Interviews, observations, document analysis | 7 stage | CATWOE, Rich picture, PAM, Root definition, Comparison of real world and PAM | Involved as informants; Healthcare professionals, Healthcare managers, Service users/representatives, Staff from other sectors | N/A | High |
| Vandenbroeck et al 2014[[40](#_ENREF_40)] | Policy | Policy improvement | Problem structuring and proposing improvements | Focus groups, workshop, literature review | Part of the method | Rich picture, PAM, Root definition | Involved in SSM; Healthcare professionals, Service users/representatives, Policy makers/administrators, representatives from other sectors | N/A | Medium |
| Pentland et al 2014[[41](#_ENREF_41)] | Mental health | Describe/improve knowledge management system | Problem structuring, proposing and implementing improvements | Focus groups | 4 activity model | Rich picture, PAM, Root definition, PQR | Involved in SSM; Healthcare professionals, R&D staff, Administrative/support staff | Substantial changes were made to the ways in which the teams acquired, stored and shared information about research, including how it was used and shared with external partners. | High |
| Cardoso-Grilo et al 2019[[42](#_ENREF_42)] | Policy | Healthcare workforce planning | Problem structuring and determining objectives for modelling/planning | Literature review, administrative data | Combined with other method | CATWOE | No participants, used administrative data | N/A | High |
| Gasson et al 2012[[43](#_ENREF_43)] | Hospital | Information system improvement | Problem structuring and proposing improvements | Interviews, observations | Combined with other method | CATWOE, PAM, Root definition, Comparison of real world and PAM | Involved in SSM; Healthcare professionals, Healthcare managers, Administrative/support staff | N/A | High |
| Holm et al 2013[[44](#_ENREF_44)] | Hospital | Care process improvement | Problem structuring, proposing and implementing improvements | Interviews, observations, workshop | Combined with other method | PAM, Root definition, Comparison of real world and PAM | Involved in SSM; Healthcare professionals, Healthcare managers, Administrative/support staff | Several improvements were implemented in the central surgery unit including the implementation of more flexibility to the duration of working shifts, which resulted in fewer procedures being cancelled due to expected overtime. | High |
| Le Fevre et al 1986[[45](#_ENREF_45)] | Hospital | Information system development/improvement | Problem structuring | Interviews | 9 stage | CATWOE, Rich picture, PAM, Root definition, Comparison of real world and PAM | Involved as informants; Healthcare managers, not clear | N/A | Low |
| Prybutok et al 2017[[46](#_ENREF_46)] | Public health | Identify important factors in millennial eHealth marketing | Problem structuring | Focus groups, literature review | New adapted version | Not stated | Involved as informants; Service users/representatives | N/A | High |
| O'Meara 2003[[47](#_ENREF_47)] | Community allied health | Describe and analyse healthcare models | Describing or understanding models of care or processes in healthcare | Interviews, focus groups, observations, survey, literature review | 4 activity model | Rich picture, PAM, Comparison of real world and PAM | Not stated; Healthcare professionals, Healthcare managers | N/A | Low |
| Adamides et al 2001[[48](#_ENREF_48)] | Blood collection establishments | Development of a common program of blood collection establishments in the European Union member states to increase safe blood management | Problem structuring and proposing improvements | Survey | 4 activity model | CATWOE, PAM, Root definition, Comparison of real world and PAM | Involved as informants; Healthcare professionals, Policy makers/administrators | N/A | Medium |
| Holm et al 2011[[49](#_ENREF_49)] | Hospital | Care process improvement | Problem structuring | Interviews, observations, workshop | 7 stage | CATWOE, Rich picture, PAM, Root definition, Comparison of real world and PAM, PQR | Involved in SSM; Healthcare professionals | N/A | High |

*Performance measurement model (adapted from the three Es)

**All references included in the final review**

1. Connell NA, Goddard AR, Philp I, Bray J. Patient-centred performance monitoring systems and multi-agency care provision: a case study using a stakeholder participative approach. Health Serv Manage Res. 1998;11(2):92-102.

2. Kalim K, Carson E, Cramp D. An illustration of whole systems thinking. Health Serv Manage Res. 2006;19(3):174-85.

3. Clarke CI, Wilcockson J. Professional and organizational learning: analysing the relationship with the development of practice. J Adv Nurs. 2001;34(2):264-72.

4. Darzentas J, Spyrou T. Information systems for primary health care: the case of the Aegean islands. Eur J Inf Syst. 1993;2(2):117-27.

5. Emes M, Smith S, Ward S, Smith A, Ming T. Care and Flow: Using Soft Systems Methodology to understand tensions in the patient discharge process. Health Syst. 2017;6(3):260-78.

6. Kotiadis K. Using soft systems methodology to determine the simulation study objectives. J Simul. 2007;1(3):215-22.

7. Kotiadis K, Tako AA, Rouwette E, Vasilakis C, Brennan J, Gandhi P, et al. Using a model of the performance measures in Soft Systems Methodology (SSM) to take action: a case study in health care. J Oper Res Soc. 2013;64(1):125-37.

8. Kotiadis K, Tako AA, Vasilakis C. A participative and facilitative conceptual modelling framework for discrete event simulation studies in healthcare. J Oper Res Soc. 2014;65(2):197-213.

9. Sinclair E, Radford K, Grant M, Terry J. Developing stroke-specific vocational rehabilitation: A soft systems analysis of current service provision. Disabil Rehabil. 2014;36(5):409-17.

10. Wells JS. Discontent without focus? An analysis of nurse management and activity on a psychiatric in-patient facility using a 'soft systems' approach. J Adv Nurs. 1995;21(2):214-21.

11. Thomas L, French B, Burton C, Sutton C, Forshaw D, Dickinson H, et al. Evaluating a systematic voiding programme for patients with urinary incontinence after stroke in secondary care using soft systems analysis and Normalisation Process Theory: Findings from the ICONS case study phase. Int J Nurs Stud. 2014;51(10):1308-20.

12. Torlak N, Muceldili B. Soft systems methodology in action: The example of a private hospital. Syst Pract Action Res. 2014;27(4):325-61.

13. Luckett S, Grossenbacher K. A Critical Systems Intervention to Improve the Implementation of a District Health System in KwaZulu-Natal. Syst Res Behav Sci. 2003;20(2):147-62.

14. Brown AD. Developing purchasing strategy: A case study of a District Health Authority using soft systems methodology. Health Serv Manage Res. 1997;10(1):58-68.

15. Burgoyne JG, Brown DH, Hindle A, Mumford MJ. A Multi-disciplinary Identification of Issues Associated with 'Contracting' in Market-oriented Health Service Reforms. Br J Manag. 1997;8(1):39-49.

16. Carr SM, Clarke CL, Molyneux J, Jones D. Facilitating participation: A health action zone experience. Prim Health Care Res Dev. 2006;7(2):147-56.

17. Carr SM, Lhussier M, Reynolds J, Hunter DJ, Hannaway C. Leadership for health improvement--implementation and evaluation. J Health Organ Manag. 2009;23(2):200-15.

18. Carter B, Whittaker K, Sanders C. Evaluating a telehealth intervention for urinalysis monitoring in children with neurogenic bladder. J Child Health Care. 2019;23(1):45-62.

19. Clarke CL, Wilcockson J. Seeing need and developing care: exploring knowledge for and from practice. Int J Nurs Stud. 2002;39(4):397-406.

20. Cook G, Gerrish K, Clarke C. Decision-making in teams: Issues arising from two UK evaluations. J Interprof Care. 2001;15(2):141-51.

21. Crowe S, Brown K, Tregay J, Wray J, Knowles R, Ridout DA, et al. Combining qualitative and quantitative operational research methods to inform quality improvement in pathways that span multiple settings. BMJ Qual Saf. 2017;26(8):641-52.

22. Fennessy G. Knowledge management in evidence-based healthcare: issues raised when specialist information services search for the evidence. Health Informatics J. 2001;7(1):4-7.

23. Gibb CE, Morrow M, Clarke CL, Cook G, Gertig P, Ramprogus V. Transdisciplinary working: Evaluating the development of health and social care provision in mental health. J Ment Health. 2002;11(3):339-50.

24. Gillies AC, Galloway J. Can soft systems methodology identify socio-technical barriers to knowledge sharing and management?: A case study from the UK national health service. Int J Knowl Manag. 2008;4(4):90-111.

25. Gillies AC, Patel I. IT and the NHS: Investigating different perspectives of IT using soft systems methodology. Stud Ethics Law Technol. 2009;3(2).

26. Hales DN, Chakravorty SS. Creating high reliability organizations using mindfulness. J Bus Res. 2016;69(8):2873-81.

27. Hindle T. Developing GP monitoring systems guided by a soft systems approach. Health Serv Manage Res. 1995;8(4):259-67.

28. Hindle T, Checkland P, Mumford M, Worthington D. Developing a methodology for multidisciplinary action research: A case study. J Oper Res Soc. 1995;46(4):453-64.

29. Hodges S, Ferreira K, Israel N. "If we're going to change things, it has to be systemic:" Systems change in children's mental health. Am J Community Psychol. 2012;49(3-4):526-37.

30. Kotiadis K, Mingers J. Combining PSMs with hard OR methods: the philosophical and practical challenges. J Oper Res Soc. 2006;57(7):856-67.

31. Lauri S. Using a computer simulation program to assess the decision-making process in child health care. Comput Nurs. 1992;10(4):171-7.

32. Lehaney B, Clarke SA, Paul RJ. A case of an intervention in an outpatients department. J Oper Res Soc. 1999;50(9):877-91.

33. Mukotekwa C, Carson E. Improving the discharge planning process: A systems study. J Res Nurs. 2007;12(6):667-86.

34. Newell K, Corrigan C, Punshon G, Leary A. Severe asthma: emergency care patient driven solutions. Int J Health Care Qual Assur. 2017;30(7):628-37.

35. O'Meara P. Would a prehospital practitioner model improve patient care in rural Australia? Emerg Med J. 2003;20(2):199-203.

36. Price M, Lau FY. Provider connectedness and communication patterns: extending continuity of care in the context of the circle of care. BMC Health Serv Res. 2013;13(309).

37. Reed J, Inglis P, Cook G, Clarke C, Cook M. Specialist nurses for older people: implications from UK development sites. J Adv Nurs. 2007;58(4):368-76.

38. Unertl KM, Weinger MB, Johnson KB, Lorenzi NM. Describing and modeling workflow and information flow in chronic disease care. J Am Med Inform Assoc. 2009;16(6):826-36.

39. Wells JSG. Hospital-based industrial therapy units and the people who work within them - An Irish case analysis using a soft-systems approach. J Psychiatr Ment Health Nurs. 2006;13(2):139-47.

40. Vandenbroeck P, Dechenne R, Becher K, Eyssen M, Van den Heede K. Recommendations for the organization of mental health services for children and adolescents in Belgium: Use of the soft systems methodology. Health Policy. 2014;114(2-3):263-8.

41. Pentland D, Forsyth K, Maciver D, Walsh M, Murray R, Irvine L. Enabling integrated knowledge acquisition and management in health care teams. Knowl Manag Res Pract. 2014;12(4):362-74.

42. Cardoso-Grilo T, Monteiro M, Oliveira MD, Amorim-Lopes M, Barbosa-Póvoa A. From problem structuring to optimization: A multi-methodological framework to assist the planning of medical training. Eur J Oper Res. 2019;273(2):662-83.

43. Gasson S. Analyzing key decision-points: problem partitioning in the analysis of tightly-coupled, distributed work-systems. IJITSA. 2012;5(2):57-83.

44. Holm LB, Dahl FA, Barra M. Towards a multimethodology in health care–synergies between Soft Systems Methodology and Discrete Event Simulation. Health Syst. 2013;2(1):11-23.

45. Le Fevre A, Pattison E. Planning for hospital information systems using the Lancaster soft systems methodology. Aust Comput J. 1986;18(4):180-5.

46. Prybutok G, Harun A, Prybutok V. eHealth marketing to millennials: a view through a systemigram. Int J Electron Healthc. 2017;9(4):319-38.

47. O'Meara P. The prehospital community-volunteer model has a place in rural Australia. JEPHC. 2003;1(1-2).

48. Adamides E, Maniatis A. A systems study for a European community program on inspection and accreditation of blood collection establishments. Syst Pract Action Res. 2001;14(5):575-96.

49. Holm LB, Dahl FA. Using soft systems methodology as a precursor for an emergency department simulation model. OR insight. 2011;24(3):168-89.
